# A Narrative Review of Anterior Cruciate Ligament Reconstruction Graft Choice and Its Influence on Return to Sport

**DOI:** 10.1007/s12178-026-10061-8

**Published:** 2026-10-21

**Authors:** Garrett R. Jackson, Justin T. Childers, Colton C. Mowers, Jaxon S. Boley, Patrick Ryan, Steven F. DeFroda, Clayton Nuelle

**Affiliations:** 1 Department of Orthopedic Surgery, University of Missouri, 1100 Virginia Avenue, Columbia, MO 65201 USA; 2 Department of Orthopedic Surgery, Orlando Health Jewett Orthopedic Institute, Orlando, FL USA; 3 Department of Surgery, University of Minnesota, Minneapolis, MN USA; 4 American University of the Caribbean School of Medicine, Miramar, FL USA; 5 Mizzou Joint Preservation Center, University of Missouri, Columbia, MO USA

**Keywords:** Anterior cruciate ligament reconstruction, Return to sport, Graft selection, Autograft, Allograft, Quadriceps tendon

## Abstract

**Purpose of Review:**

Anterior cruciate ligament (ACL) rupture is among the most common knee injuries in active individuals and can lead to persistent instability, secondary intra-articular damage, and an elevated risk of osteoarthritis. Because return to sport (RTS) is a major goal for most active patients and graft selection remains one of the most debated aspects of ACL reconstruction, this narrative review summarizes the contemporary evidence on how graft choice influences RTS, focusing on the four most common grafts: bone–patellar tendon–bone (BTB), hamstring tendon (HT), quadriceps tendon (QT), and allograft.

**Recent Findings:**

RTS rates range from ~ 65% to 73% at three years and are higher (83–90%) in elite and high-school athletes. In young, active patients, autografts are associated with higher RTS and lower revision rates than allografts, and high-level evidence—including randomized-trial meta-analyses—has not established the superiority of any single autograft. BTB offers reliable bone-to-bone healing and low revision rates; HT reduces donor-site morbidity and anterior knee pain but has variable diameter and possible flexion weakness; and QT is a versatile option with favorable biomechanics, low donor-site morbidity, and encouraging RTS data, including in skeletally immature athletes.

**Summary:**

RTS is one of several important outcomes after ACL reconstruction, alongside long-term joint health and reinjury prevention. For RTS, the literature supports autograft over allograft in young, active patients while identifying no universally superior autograft. Graft selection should be individualized to patient age, sport, activity level, anatomy, and reinjury risk; the senior author favors QT or BTB autografts for high-demand athletes.

## Introduction

Anterior cruciate ligament (ACL) injuries occur primarily in young and active individuals, often in those participating in pivoting and contact sports or activities that involve rapid deceleration and changes of direction [1–4]. The incidence of ACL injury in younger individuals is rising with earlier and more intensive sports participation, and these young, active patients are also at particularly elevated risk for reinjury [2, 5, 6]. As return to sport (RTS) is a main goal for most athletes, surgical stabilization in the form of ACL reconstruction is the most common option for this injury [7].

Recent studies with large sample sizes [8, 9] report a range of RTS rates, from 65% to 73.2% overall at 3 years, with approximately half of patients returning to their pre-injury level of performance [7, 10]. Investigations of subgroups such as high-school-aged and elite professional athletes report higher rates, from 83% to over 90% [11–13]. While factors such as male sex, younger age, and a shorter interval between injury and surgery have been shown to positively influence RTS, evidence on the influence of graft choice on RTS remains conflicting, and controversy persists regarding the optimal graft [14].

In the ACL Study Group’s 2021 biennial survey of global trends in ACL reconstruction, Sherman et al. reported that the most common graft choices in primary ACL reconstruction were the hamstring tendon (HT), bone–patellar tendon–bone (BTB), quadriceps tendon (QT), and allograft, in decreasing order of popularity [15]. BTB and QT grafts were the most prevalent graft preferences for revision reconstruction [15]. In revision cases in athletes, comparisons of BTB and QT grafts suggest similar mid- to long-term clinical and functional outcomes; QT autografts may afford faster RTS and better short-term (< 1 year) clinical improvement; however, these differences normalize at mid- to long-term follow-up [16].

The purpose of this review is to present the relevant considerations and evidence for each major graft choice, to critically assess studies comparing graft choices, and to offer recommendations on the optimal application of each option with respect to RTS.

## Graft Options

### Patellar Tendon

**Fig. 1:**
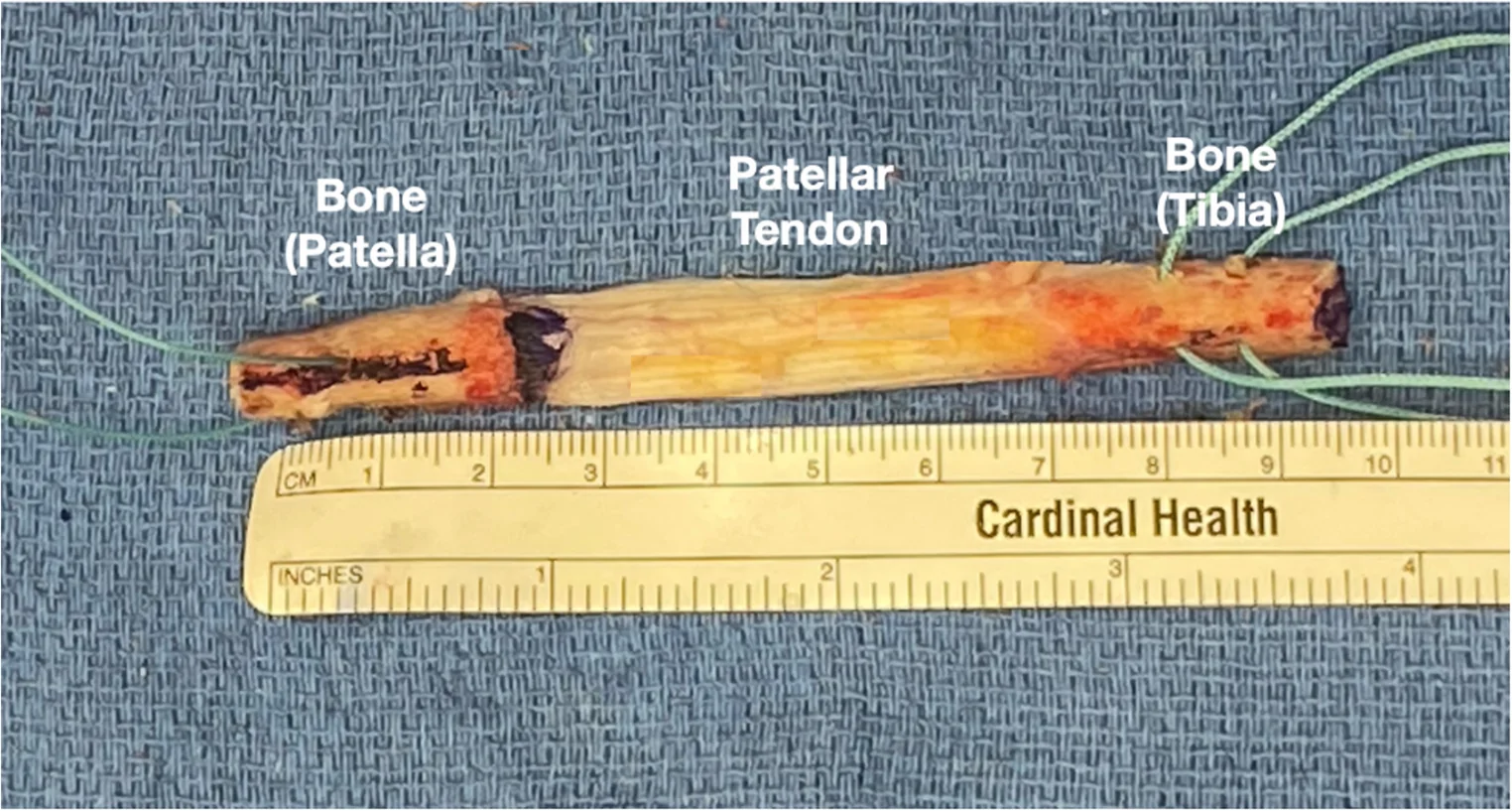
Gross photograph of a bone–patellar tendon–bone (BTB) autograft prior to ACL reconstruction

BTB grafts are harvested from the central third of the patellar tendon, with bone blocks from the inferior pole of the patella proximally and the tibial tubercle distally (Fig. 1). The central third is the preferred harvest site, as it demonstrates superior maximum load, stress, and stiffness compared with lateral and medial hemi-BTB grafts [17]. Increasing graft length correlates with greater tensile strength, although it remains unclear whether a larger cross-sectional area directly increases graft strength [18, 19].

Although graft choice for ACLR remains debated and depends on each surgeon’s experience and preference, BTB grafts have historically been considered the gold standard because of favorable clinical outcomes [20, 21], low revision rates [20, 22, 23], and the advantage of bone-to-bone healing [24]. In terms of biologic incorporation, maximum pull-out strength is achieved faster with bone-to-bone healing (6–8 weeks) than with tendon-to-bone healing (8–12 weeks) [25]; this earlier incorporation may permit a more aggressive early rehabilitation and loading progression with BTB grafts. Patellar tendon autografts also exhibit increased magnetic resonance imaging (MRI) signal during the first year, in contrast to the low signal seen in HT reconstructions, suggesting superior early graft maturity that may allow safer RTS [26]. Of the surgeons polled at an ISAKOS consensus meeting on return to soccer following ACLR, approximately 70% favored BTB grafts in patients participating in pivoting sports to allow more aggressive rehabilitation and faster return to soccer [26].

Conversely, BTB harvest carries inherent disadvantages, most notably patellar fracture and persistent anterior knee pain related to donor-site morbidity [27]. Contemporary data indicate that patellar fracture is uncommon: a large propensity-matched cohort reported an incidence of only 0.21% (12 of 5,770 reconstructions) after BTB harvest [28], and a recent biomechanical study by Partan et al. [29] found the likelihood of patellar fracture during harvest to be very low for grafts up to 25 mm in length, diminishing one of the chief concerns surrounding this graft. It is nonetheless often hypothesized that BTB may adversely affect performance in sports requiring frequent kneeling, such as wrestling and judo [10, 30]. Careful consideration is also warranted in skeletally immature patients, given the potential for physeal damage during harvest and fixation [10].

Regarding durability, multiple studies have demonstrated consistently low failure rates with BTB autografts, particularly in young athletic populations. In a systematic review of women ≤ 25 years of age, Etzel et al. [20] reported an overall graft-failure rate of only 6%, supporting BTB use in a population at high risk for reinjury. Likewise, large Scandinavian registry studies have shown lower revision rates with BTB than with HT autografts, particularly among younger, highly active patients [22, 31]. The favorable survivorship of BTB grafts is thought to reflect a combination of bone-to-bone healing, predictable graft size, and restoration of rotational stability [32]. Collectively, these findings have contributed to BTB remaining one of the preferred graft choices for competitive athletes and patients in pivoting sports.

Supplemental techniques are increasingly used to optimize outcomes when a BTB graft is selected. Matava et al. [33] identified suture-tape augmentation as a means to increase BTB graft stiffness and load to failure, and this technique has demonstrated improved biomechanical performance compared with interference-screw fixation alone, particularly for BTB grafts [34, 35]. Procedures aimed at improving rotational stability have also gained interest; lateral extra-articular tenodesis (LET) is an effective addition to ACLR in young, high-demand athletes and is indicated in patients with high-grade rotatory laxity, revision reconstruction, generalized ligamentous laxity, or a hyperextension profile, reducing anterolateral rotation and the incidence of graft failure [36–38].

From an RTS perspective, BTB autografts remain among the most extensively studied options. A systematic review by DeFazio et al. [9] of more than 2,300 athletes demonstrated an overall RTS rate of 81% after BTB reconstruction compared with 70.6% for HT autografts, although rates of return to preinjury performance were similar (BTB 50.0% vs. HT 48.5%). More recent randomized-trial meta-analyses have shown no significant difference in return to baseline sport between BTB and HT grafts, suggesting that although BTB may facilitate a high overall RTS rate, graft selection should be individualized to patient-specific factors rather than RTS expectations alone [39].

### Hamstring Tendon

**Fig. 2:**
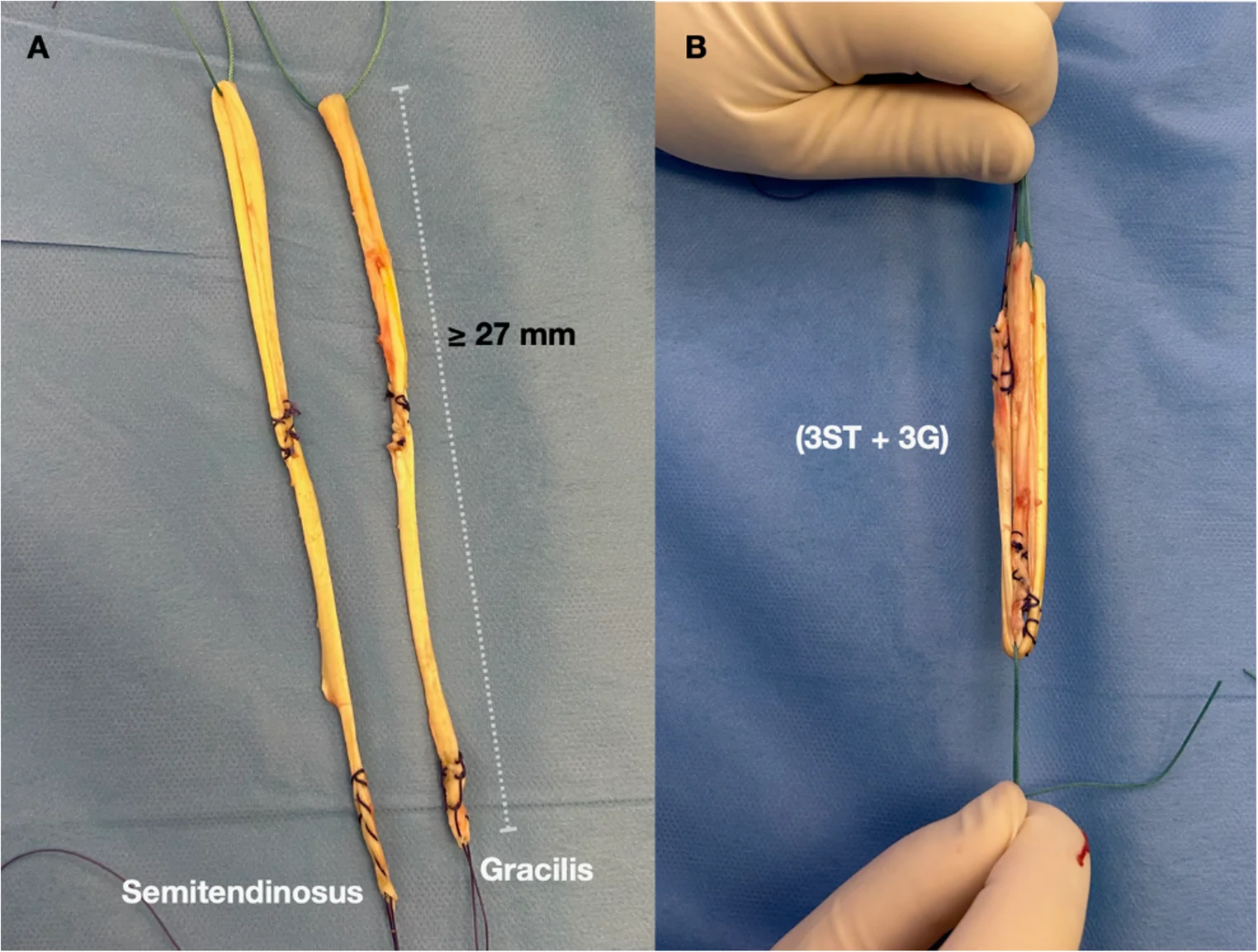
Gross photograph of semitendinosus and gracilis tendons harvested from a patient (**A**) and configured into a 6-strand graft for ACL reconstruction (**B**). ST, semitendinosus; G, gracilis

HT grafts are typically harvested from the semitendinosus and gracilis tendons at their tibial insertion [32]. Graft size is an especially important predictor of HT autograft success [40–43]; the tendons are therefore commonly folded into a quadruple-stranded construct, and longer tendons allow quintuple- or sextuple-stranded configurations (Fig. 2) [32]. Because the diameter of a quadruple-stranded HT graft varies among patient demographics, a 6- or 8-stranded construct may be required to achieve an adequate diameter in some patients [44]. To optimize the graft’s structural properties (load to failure and stiffness), each strand must be equally tensioned [45].

Biomechanically, HT grafts show an ultimate load to failure similar to that of BTB and QT grafts (*p* = 0.951) and a greater elastic modulus (*p* = 0.016), but a lower cross-sectional area than both (*p* < 0.001) and less stiffness than QT grafts (*p* = 0.004) [46]. Overall, these findings indicate less favorable structural properties for HT grafts relative to the other autografts used in ACLR.

Reported clinical advantages of HT autograft include a smaller harvest incision, decreased donor-site morbidity [32], and lower postoperative anterior knee pain and quadriceps dysfunction than BTB or QT grafts [47]. As with BTB grafts, HT grafts can yield low revision rates in some cohorts [23]; however, large registry and meta-analytic studies have reported higher failure rates for HT than BTB grafts, particularly among younger, highly active patients [22, 31]. Use in specific subgroups—including female patients and those with genu recurvatum—may further increase failure rates [48, 49]. Inferences from large retrospective databases should be interpreted with caution, especially regarding RTP decisions [20, 22, 31].

Several disadvantages temper the use of HT grafts. Harvest may cause persistent weakness in knee flexion [50], so the graft is best avoided in athletes who rely heavily on hamstring function, such as sprinters and soccer players [10]. In addition, because of the risk of inadvertent medial collateral ligament (MCL) injury during harvest and the role of the hamstrings in dynamic resistance to valgus stress, an HT graft may be suboptimal in the setting of concomitant medial instability [51].

In a prospective cohort, Stropnik et al. [52] found that patients who perceived their knee as normal at 6 months after HT reconstruction had better thigh-muscle neuromuscular properties, lower hamstring stiffness, and higher RTS at 12 months than those who perceived their knee as sub-normal. These findings underscore the importance of restoring hamstring flexibility, range of motion, and neuromuscular control during rehabilitation after HT reconstruction.

Overall, HT autografts remain an excellent option for many patients, particularly those wishing to minimize anterior knee pain and donor-site morbidity [32]. When RTS is the primary objective, however, surgeons should weigh the potential for persistent hamstring weakness, variability in graft diameter, and the modestly lower RTS rates reported for HT relative to BTB grafts in several syntheses [9, 14, 31, 39]. With graft-specific rehabilitation that restores hamstring strength and neuromuscular control (discussed below) and satisfaction of objective RTS criteria, carefully selected patients can nonetheless achieve excellent functional outcomes and successful RTS after HT reconstruction [47].

### Quadriceps Tendon

**Fig. 3:**
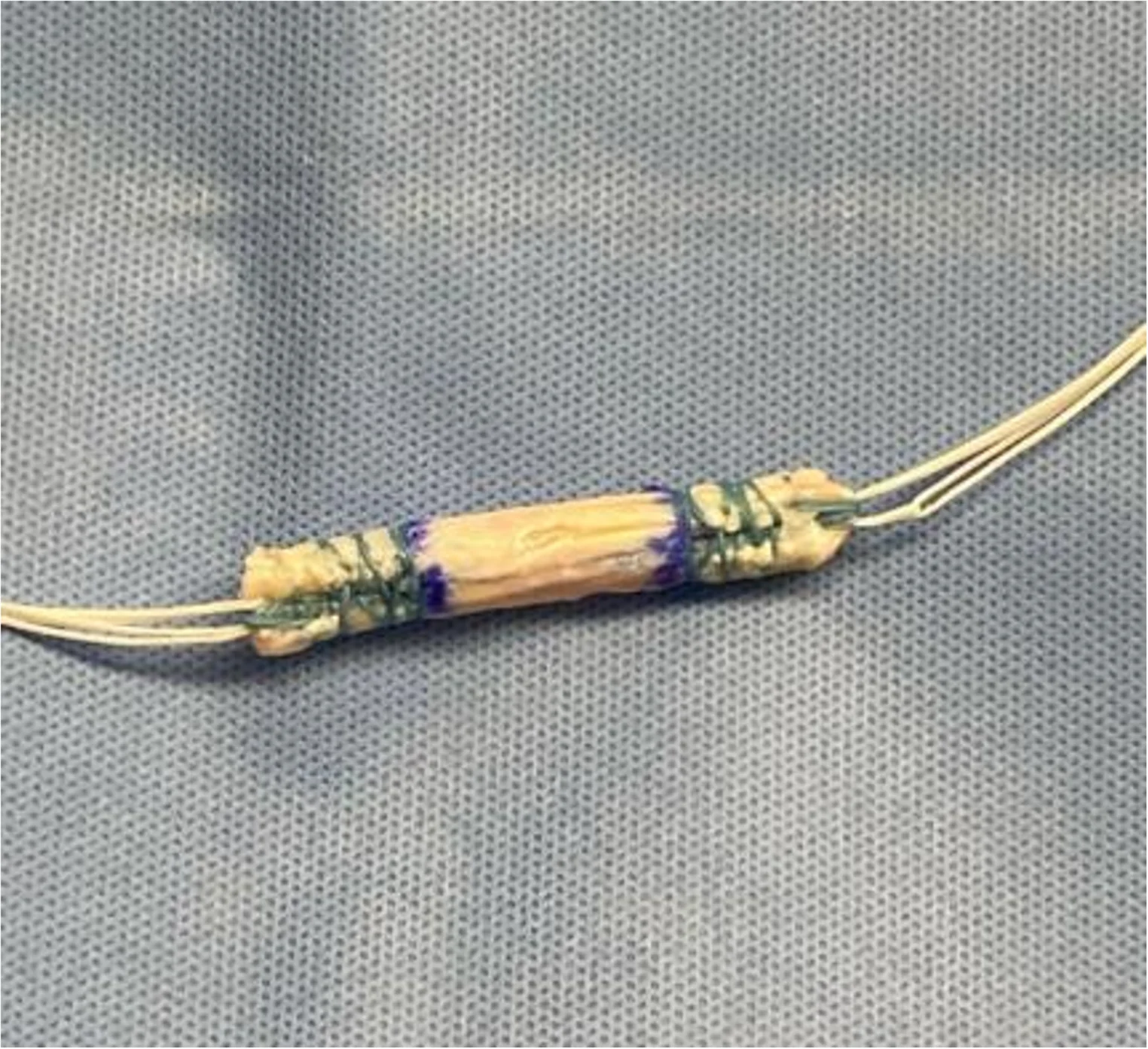
Gross photograph of an all-soft-tissue quadriceps tendon autograft prior to ACL reconstruction

The use of QT grafts has gained popularity in recent years, roughly doubling among young European surgeons over a recent five-year period [53]. QT grafts can be harvested with or without a patellar bone plug (Fig. 3) and as full- or partial-thickness grafts; full-thickness harvest incorporates the entire tendon, whereas partial-thickness harvest preserves the deep layer and leaves a greater proportion of native tendon behind [54, 55]. A principal advantage of the QT graft is the large volume of native tendon remaining after harvest [54, 56]. In a morphological analysis, Xerogeanes et al. [54] showed that the quadriceps tendon contains a greater volume of native tissue than the hamstring or patellar tendons. This permits a reproducible, predictable graft with substantially greater intra-articular volume than a patellar tendon graft of similar width. Biomechanically, QT grafts provide a mean cross-sectional area nearly twice that of BTB grafts, with higher load to failure and stiffness [57].

Indications for a QT graft include primary reconstruction in high-demand patients as well as revision settings and skeletally immature patients [58]. Relative to a patellar tendon graft, the QT provides greater intra-articular graft volume and collagen content [17, 54, 56]. Several disadvantages parallel those of BTB grafts. Quadriceps strength is reduced in the early postoperative period and, to a lesser degree, at long-term follow-up, particularly with full-thickness grafts [59]. Persistent anterior knee pain and postoperative hematoma may occur, although anterior knee pain is less common than after BTB reconstruction. Patellar fracture is an uncommon complication when a patellar bone plug is harvested [55, 60]. Notably, a systematic review and meta-analysis found no significant difference in outcomes between all-soft-tissue QT grafts and QT grafts with a bone plug [61].

Recent studies have shown favorable outcomes and low rates of donor-site morbidity with QT grafts at follow-up of up to at least 7.5 years [59, 62]. QT reconstruction does, however, carry a learning curve: higher revision rates for QT than for BTB or HT grafts were reported in the Danish Knee Ligament Registry, and this difference was confined to centers performing fewer than 100 QT cases over 8 years [63].

In a 5-year follow-up study, Galan et al. [60] reported a 73% RTS rate after reconstruction with an all-inside full-thickness QT graft with a bone block, at a mean RTS time of 8.2 months (range, 6.5–11 months). Vaughn et al. [64] observed an 86% RTS rate at a mean 2.8-year follow-up. In pediatric patients, a recent systematic review by Zakharia et al. [65] reported RTS rates of 88.9% to 91.6% after QT reconstruction.

### Allograft

Allograft tissue is typically sourced from the patellar, quadriceps, hamstring, Achilles, or tibialis (anterior or posterior) tendons [66]. Allograft is used uncommonly in primary ACLR in patients younger than 30 years and is generally reserved for older, less active patients, because registry and cohort studies consistently show an increased risk of revision with allograft—particularly in younger patients and those participating in high-impact or pivoting sports [67–71]. For example, large integrated health-system registry data have demonstrated a significantly higher adjusted revision risk for soft-tissue allografts than for autografts [69, 70]. Allograft also carries a small but non-zero risk of disease transmission, and graft healing and maturation are slower than with autograft [72]. Its advantages include the absence of donor-site morbidity, shorter operative time, and avoidance of the extensor-mechanism morbidity associated with QT or BTB harvest and of the effect on medial knee stabilizers that can follow HT harvest [66]. Consequently, allograft may be a reasonable option for individuals ≥ 50 years of age seeking a return to lower-demand activity [73–75], in whom a delayed RTS of at least 12 months is nonetheless recommended to allow adequate incorporation [76].

In young, athletic populations, RTS after allograft reconstruction is less favorable than after autograft, primarily because of higher graft-failure rates [67–71]. Daruwalla et al. [77] evaluated NCAA Division I football players and found a higher return-to-play rate with autograft than allograft (84.9% vs. 69.9%; *p* = 0.045). These findings support the recommendation that autograft remain the preferred option for patients intending to return to pivoting or collision sports [67–71, 76]. In older or lower-demand patients, by contrast, graft choice appears to have minimal influence on outcomes, and allograft is a reasonable choice [66, 73].

## Comparative Studies

The higher graft-failure rate of allograft in young, active patients is paralleled by less favorable RTS, as discussed above [77]. Among autografts, comparative evidence is most abundant for BTB versus HT grafts.

A 2015 meta-analysis by Xie et al. [32], restricted to 4-strand HT autografts, found a higher rate of return to preinjury level with BTB (61% vs. 51%; *p* = 0.03). A later systematic review of 2,348 patients by DeFazio et al. [9] similarly reported RTS of 81% for BTB versus 70% for HT. The highest-level evidence, however, comes from Bergeron et al. [39], who performed a meta-analysis of randomized controlled trials comprising more than 3,000 patients and found no significant difference between BTB and HT in the relative risk of returning to baseline sport; the authors concluded that no firm recommendation on an optimal graft can be made when RTS is the primary metric. Graft choice may nevertheless influence the rehabilitation timeline: in a secondary analysis of the ACL-SPORTS trial, Smith et al. [75] found that athletes with BTB grafts were slower than those with HT or soft-tissue allograft to reach a quadriceps strength index of 80% and 90%—a common objective RTS criterion.

Comparative long-term data for QT autografts, including RTS, remain relatively sparse [55]. In a prospective study of 60 athletes (baseline Tegner score ≥ 6), Barié et al. [78] found similar return-to-preinjury-sport levels after primary reconstruction with a QT–patellar bone graft versus a BTB graft. In the revision setting, the same group reported that QT and HT autografts yielded similar rates of return to preinjury sport [79]. Consistent with these findings, a systematic review by Kay et al. [80] found that graft choice (BTB, HT, or QT) had no significant influence on RTS rates after revision ACL reconstruction.

## Rehabilitation and Return-to-Sport Assessment

Rehabilitation should be tailored to the graft, because donor-site morbidity differs by harvest. After BTB reconstruction, early rehabilitation emphasizes restoration of terminal extension and quadriceps function while protecting the patellofemoral joint and managing anterior knee pain; because bone-to-bone healing achieves fixation strength earlier than soft-tissue healing, a comparatively more aggressive early loading progression may be tolerated [25]. After HT reconstruction, emphasis shifts to recovery of hamstring (knee-flexion) strength and neuromuscular control, given the potential for persistent flexion weakness and altered hamstring stiffness [50, 52]. After QT reconstruction, the early quadriceps strength deficit is the principal rehabilitation target, and progression is often more graduated in the first postoperative months [59]. Across grafts, contemporary clinical practice guidelines advocate criterion-based rather than time-based progression [1, 7].

Regardless of graft, the decision to return to sport should be criterion-based and informed by objective functional testing rather than time alone. Commonly used measures include limb-symmetry indices for quadriceps and hamstring strength and a hop-test battery, together with assessment of psychological readiness using the ACL–Return to Sport after Injury (ACL-RSI) scale [81]. Meeting objective discharge criteria before RTS is strongly associated with lower reinjury risk: athletes who failed to meet six clinical discharge criteria had a fourfold higher risk of graft rupture [82], and passing a return-to-sport test battery combined with delaying RTS until at least 9 months reduced reinjury by more than 80% in the Delaware–Oslo cohort [83]. Because graft choice influences the trajectory of strength recovery—for example, slower quadriceps recovery after BTB and QT grafts and slower hamstring recovery after HT grafts—these graft-specific deficits should be explicitly targeted and objectively confirmed before clearance [75]. Reported mean RTS times generally range from approximately 8 to 12 months and should be guided by criterion achievement rather than fixed timelines [60].

## Senior Author’s Preferences

Graft selection remains debated, and no single graft is universally superior for RTS across all patients; sex, size, age, sport, activity level, and expectations should all inform the choice [14, 30]. For primary reconstruction in high-demand athletes, the senior author generally prefers a BTB or all-soft-tissue QT autograft, favoring these grafts for their predictable size and favorable biomechanical and clinical profiles [46, 57]. BTB is particularly useful when rapid, robust incorporation is desired—for example, in collision-sport athletes and patients with marked rotatory laxity—reflecting its bone-to-bone healing and low revision rates [20, 22, 25]. The QT is favored as a versatile primary option and in the revision setting, especially after a prior BTB graft, as it preserves the patellar tendon while providing ample graft tissue [55, 61].

In skeletally immature patients, the senior author prefers an all-soft-tissue QT graft to avoid bone plugs crossing open physes while still providing adequate tissue; reported pediatric outcomes with this graft have been favorable, although comparative long-term data remain limited [58, 65]. HT autografts and allografts are used more selectively—HT when minimizing anterior knee pain and donor-site morbidity is a priority, but with caution in patients who rely on hamstring function or have medial instability, and allograft principally in older, lower-demand patients or when autograft tissue is unavailable [10, 51, 73]. Ultimately, graft choice is individualized to the patient rather than dictated by any single factor.

## Limitations

As a narrative rather than a systematic review, this article did not use a predefined search protocol or formal quality assessment, and the studies discussed are clinically heterogeneous, with variable definitions of return to sport and follow-up. Much of the comparative evidence derives from registry and retrospective data, and the senior author’s preferences reflect expert opinion.

## Conclusions

When RTS is considered a primary outcome, the available literature supports the use of autograft tissue over allograft in young and active patients. However, current high-quality evidence does not demonstrate consistent superiority of one autograft option over another with respect to RTS rates alone. BTB grafts offer reliable bone-to-bone healing and low revision rates, HT grafts provide less donor-site morbidity and less anterior knee pain, and QT grafts have emerged as a versatile option with favorable biomechanical properties and encouraging clinical outcomes. Therefore, graft selection should be individualized according to patient age, sport, activity level, anatomic considerations, and risk of reinjury. For high-demand athletes, the authors generally favor QT or BTB autografts because of their predictable graft characteristics and favorable biomechanical and clinical outcome profiles. These recommendations reflect the current best available evidence together with expert opinion and should be interpreted in light of the limitations noted above; higher-quality comparative studies using standardized return-to-sport criteria are needed to define the optimal graft for the individual patient.

## Key References

- Bergeron JJ, Sercia QP, Drager J, Pelet S, Belzile EL. Return to Baseline Physical Activity After Bone-Patellar Tendon-Bone Versus Hamstring Tendon Autografts for Anterior Cruciate Ligament Reconstruction: A Systematic Review and Meta-analysis of Randomized Controlled Trials. Am J Sports Med. 2022;50(8):2292–2303.
  - ○ A meta-analysis of randomized controlled trials—the highest available level of evidence directly comparing bone–patellar tendon–bone and hamstring autografts—that found no significant difference in return to baseline sport. It is central to our conclusion that no single autograft is universally superior for return to sport.
- DeFazio MW, Curry EJ, Gustin MJ, et al. Return to Sport After ACL Reconstruction With a BTB Versus Hamstring Tendon Autograft: A Systematic Review and Meta-analysis. Orthop J Sports Med. 2020;8(12):2325967120964919.
  - ○ A large systematic review and meta-analysis reporting overall return-to-sport rates by graft (81% for BTB vs 70.6% for hamstring) while showing similar return to preinjury performance, which frames the practical trade-offs between the two most-studied autografts.
- Therien AD, Kouo T, Prado I, et al. No Postoperative Difference Between Bone-Block Versus All-Soft Tissue Quadriceps Tendon Autografts for Anterior Cruciate Ligament Reconstruction: A Systematic Review and Meta-analysis. Orthop J Sports Med. 2026;14(3):23259671261418051.
  - ○ A recent systematic review and meta-analysis showing no significant difference in outcomes between bone-block and all-soft-tissue quadriceps tendon grafts, supporting the versatility of the quadriceps tendon graft emphasized in this review.
- Lind M, Strauss MJ, Nielsen T, Engebretsen L. Quadriceps tendon autograft for anterior cruciate ligament reconstruction is associated with high revision rates: results from the Danish Knee Ligament Registry. Knee Surg Sports Traumatol Arthrosc. 2020;28(7):2163–2169.
  - ○ A national registry study demonstrating higher revision rates with quadriceps tendon grafts, concentrated among lower-volume surgeons. It is included to balance enthusiasm for the quadriceps tendon and to underscore the learning curve associated with this graft.
- Zakharia A, Lameire DL, Abdel Khalik H, et al. Quadriceps tendon autograft for pediatric anterior cruciate ligament reconstruction results in promising postoperative function and rates of return to sports: A systematic review. Knee Surg Sports Traumatol Arthrosc. 2022;30(11):3659–3672.
  - ○ A systematic review reporting promising function and return-to-sport rates after quadriceps tendon reconstruction in pediatric patients, informing the discussion of quadriceps tendon use in skeletally immature athletes.
- D'Ambrosi R, Ackermann J, Deichsel A, et al. Graft choice does not influence outcomes after anterior cruciate ligament reconstruction in patients aged 50 years and older: A systematic review and meta-analysis. J ISAKOS. 2026;18:101111.
  - ○ A recent meta-analysis showing that graft choice does not significantly influence outcomes in patients aged 50 years and older, reinforcing the review's central theme that graft selection should be individualized to the patient.

## Data Availability

No datasets were generated or analysed during the current study.
